# A Fast Hybrid Classification Algorithm with Feature Reduction for Medical Images

**DOI:** 10.1155/2022/1367366

**Published:** 2022-03-22

**Authors:** Hanan Ahmed Hosni Mahmoud, Abeer Abdulaziz AlArfaj, Alaaeldin M. Hafez

**Affiliations:** ^1^Department of Computer Sciences, College of Computer and Information Sciences, Princess Nourah Bint Abdulrahman University, P.O. Box 84428, Riyadh 11671, Saudi Arabia; ^2^Department of Information Systems, College of Computer and Information Sciences, King Saud University, Riyadh, Saudi Arabia

## Abstract

In this paper, we are introducing a fast hybrid fuzzy classification algorithm with feature reduction for medical images. We incorporated the quantum-based grasshopper computing algorithm (QGH) with feature extraction using fuzzy clustering technique (*C*-means). QGH integrates quantum computing into machine learning and intelligence applications. The objective of our technique is to the integrate QGH method, specifically into cervical cancer detection that is based on image processing. Many features such as color, geometry, and texture found in the cells imaged in Pap smear lab test are very crucial in cancer diagnosis. Our proposed technique is based on the extraction of the best features using a more than 2600 public Pap smear images and further applies feature reduction technique to reduce the feature space. Performance evaluation of our approach evaluates the influence of the extracted feature on the classification precision by performing two experimental setups. First setup is using all the extracted features which leads to classification without feature bias. The second setup is a fusion technique which utilized QGH with the fuzzy C-means algorithm to choose the best features. In the setups, we allocate the assessment to accuracy based on the selection of best features and of different categories of the cancer. In the last setup, we utilized a fusion technique engaged with statistical techniques to launch a qualitative agreement with the feature selection in several experimental setups.

## 1. Introduction

Cervical cancer is a deadly disease that affects many women. Medical testing technology can detect cervical cancer by performing Pap smear medical test. Pap smear test filters abnormal ailed cervical cells which leads to distinguish precancerous alteration in cervical cells [1, 2]. Color and shape alteration of the nuclei and cytoplasm can implicate the occurrence of Papilloma virus that causes cervical cancer [3, 4]. Manual Pap smear testing is slow and error-prone procedure and requires pathology experts [5, 6]. It was found that a lot of inconsistencies from the manual test can compromise the validity the Pap smear process [7]. Hundreds of patients undergo the Pap smear test every day with a lot of images to be manually analyzed. This will hinder the classification of the cells into normal or cancerous and might lead to errors [8]. Pap smear test can classify a cell into several classes including superficial and intermediate squamous as well as mild, moderate, and severe dysplasia. Also columnar and carcinoma are identified by the pear smear test. The accuracy of manual cell type detection prevails inaccurate classification as well as long diagnosis time. The quantum-based grasshopper computing algorithm (QGH) improved the ability of the standard grasshopper computing (SGC) technique.

The need for an automated detection method for cervical cell type is required. Automated segmentation methods are required to outline the cytoplasm and nucleus contours of the cell from Pap smear images. Several automated methods for Pap smear image analysis are proposed in the literature [8–12]. The authors in [8] utilized fourteen features and validated their classification using five classifiers. They emphasize on analyzing images from digital colposcopy. The research in [12] introduced a neurofuzzy classification method to identify twenty features in cervical cells. The authors in [13] proposed a computerized cell segmentation of the cervical. They also applied a classification technique on four Pap smear datasets. In [14], they utilized nine features and classified them through a support vector machine that eliminated features recursively.

Hybrid systems, that incorporate intelligence, usually integrate more than one intelligent methodology. Intelligent methodology includes fuzzy techniques, case-based reasoning, and neural networks. Hybrid systems have the ability to deal with complex problems that comprise uncertainty and high-dimensional complexity [15]. Hybrid systems are practically found in every real world problem, especially in medical applications. A filter feature selection method is a computationally fast, scalable selection method as stated in [16]. The wrapper and hybrid techniques exhibit higher performances than the filter feature selection methods. Hybrid methods commonly use supervised learning techniques and grasshopper-based intelligent methods as integral components of their feature selection. Several hybrid methods use grasshopper intelligence feature selection algorithms [17]. Other studies utilize the quantum grasshopper optimization technique by developing quantum mechanic properties which prevail better performance in the search capability [18].

The accuracy of manual cell type detection prevails inaccurate classification as well as long diagnosis time. The need for an automated detection method for cervical cell type is required. Automated segmentation methods are required to outline the cytoplasm and nucleus contours of the cell from Pap smear images.

Quantum grasshopper optimization technique has been enhanced as in the work in [19], and they proposed a local and global search policies balancing. Also, the authors in [20] enhanced the quantum algorithm by utilizing visual features choice. Global optimal is utilized to define the best feature selection and this accelerates the convergence of feature selection.

Several automated methods for Pap smear image analysis are proposed in the literature [21]. Other studies utilize the quantum grasshopper optimization technique by developing quantum mechanics properties which prevail better performance in the search capability [22]. In [23], the authors suggested a technique to classify cervical cancer eliminating segmentation parameters. They built deep feature sets, using CNN nets. First, the CNN is pertained on ordinary images and then fine-tuned on a Pap smear dataset of resampled image areas centered at the nuclei. Also, the authors in [24] enhanced the quantum algorithm by utilizing visual features choice. Global optimal is utilized to define the best feature selection and this accelerate the convergence of feature selection. The authors in [25] improved the accuracy of the quantum algorithm by utilizing chromatic features spectrum. In [26], the authors provide an ensemble transfer learning model from cervical histopathology features with satisfactory accuracy rate. Their model has a high prediction performance due to the employing of a weighted voting learning model.

Our technique, proposed in this paper, utilizes the quantum grasshopper optimization technique (QGH) as the central module of a novel hybrid approach for feature selection in images of the cervical cells produced by the Pap smear test. The QGH will be combined with the fuzzy *C*-means algorithm. Our proposed technique will enhance the choice of features by relating the QGH algorithm with the fuzzy *C*-means algorithm. The experimental results prove that proposed hybrid system entices better accuracy in the classification of cervical cells. To validate the accuracy of our technique, we used two datasets presented in [24, 25], which includes original images as well as segmented images. We used 13 geometric color and texture features to describe the Pap smear images. We pruned the features into a collection of six features. The feature pruning step preceded with the fuzzy *C*-means improves cell classification and prediction procedures.

The rest of the paper is divided as follows: cell classification in Pap smeared images are depicted in Section 2. An overview of the proposed QGH and fuzzy *C*-means method is depicted in Section 3. Similar state-of-the art classification and feature selection algorithm description are described in Section 4. Experimental results are reported in Section 5. Conclusion is demonstrated in Section 6.

## 2. Cell Classification in Pap Smeared Images

Cervical cancer is a cancer that is built in the cervical cells [14]. Pap smear test is a visual test and is considered as the main medical procedure that is utilized to diagnose the existence of Papilloma virus, which is responsible for cervical cancer. Pap smear helps in early diagnosis and can save lives before the cancer deteriorates. Pap smear classifies the cells into seven classes as depicted below.

**Figure 1:**
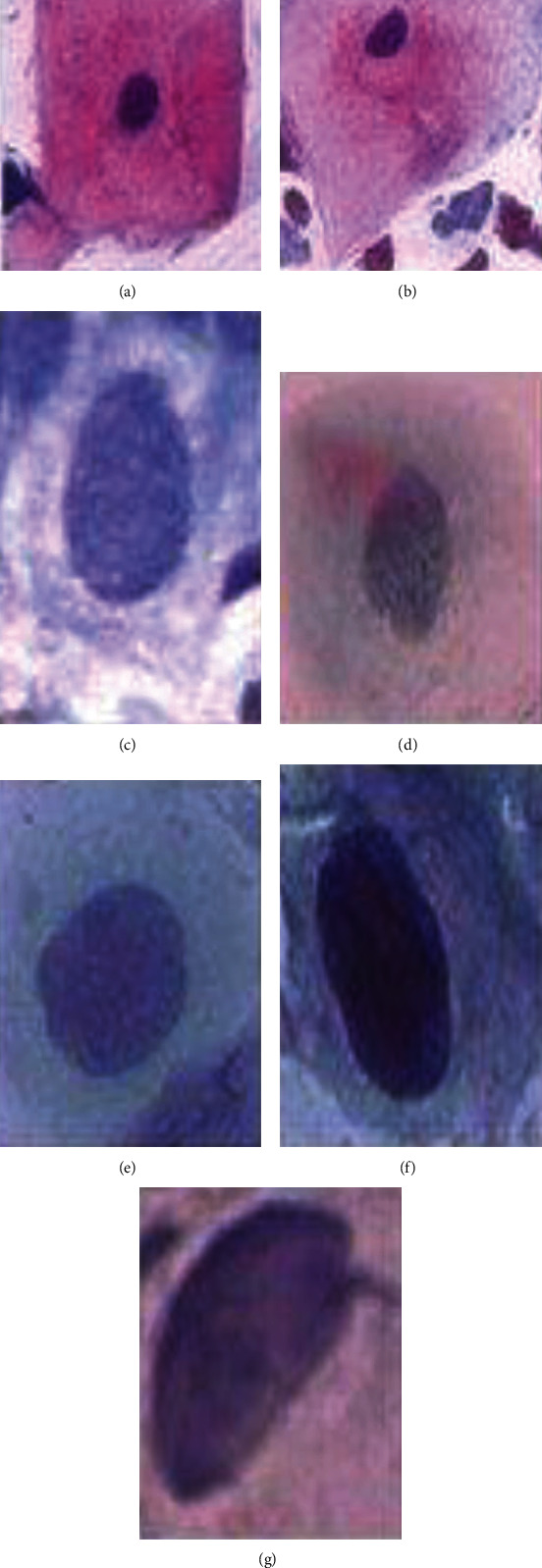
[24, 25] Pap smear image: (a) preliminary squamous, (b) intermediate, (c) columnar squamous, (d) Preliminary dysplasia, (e) moderate, (f) severe, and (g) cancer.

Dysplastic cells are abnormal cervical cells that have a precancerous state. They are allocated into four phases. The first one is mild dysplasia, which arises from the growing of the nucleus. The second one is the moderate dysplasia phase where the nucleus develops a darker color. The third one is the severe dysplasia where the size of the nucleus as well as the cytoplasm is altered, where the nucleus becomes larger and the cytoplasm becomes smaller. The fourth phase is the carcinoma in situ, where the nucleus becomes very large and becomes malignant. Cell properties help us to classify the cells as cancerous or precancerous cells. Properties such as shape, size, and morphology of the cytoplasm could lead to the cancer diagnosis due to changes in nuclear-cytoplasm ratios. Pap smear tests of cells with different color, shape, and size are present in Figure 1. Pap smear test can classify a cell into several classes including superficial and intermediate squamous as well as mild, moderate, and severe dysplasia. Also columnar and carcinoma are identified by the pear smear test. The accuracy of manual cell type detection prevails inaccurate classification as well as long diagnosis time.

### 2.1. Grasshopper Quantum Computing (QGH)

Quantum-based computing (QGH) is a paradigm that utilizes quantum mechanics to process information to enhance computing paradigm [25], especially in image processing and machine intelligence [26–28]. QGH integrates quantum computing into machine learning and intelligence applications [29]. The objective of our technique is to integrate QGH methods, specifically QGH computing into cervical cancer detection that is based on image processing [30]. The quantum-based QGH algorithm (QGH) improved the ability of the SGC technique.

The QGH surveyed many algorithms starting with the standard grasshopper computing (SGC) which imitates the grasshopper searching for food in a known space, with expected intelligence following the other bird population. There is a major restriction of one source only of food without prior knowledge of food location. The straightforward solution is to trail the one bird which found the location. Therefore, the other grasshopper will traverse the same path to the food location with no consideration of their proximity to the food location. In QGH paradigm, each solution is named a particle. The optimal solution is computed in the search space by updating the previous solution. The particles utilize fitness values and speed values to fly and follow different paths of the particles to find better solutions. The quantum-based QGH algorithm improved the ability of the SGC technique. The probability of the particle found in location *X* was computed from the quantum wave function of the particle at current location (*t*).

## 3. The Proposed Model

**Figure 2:**
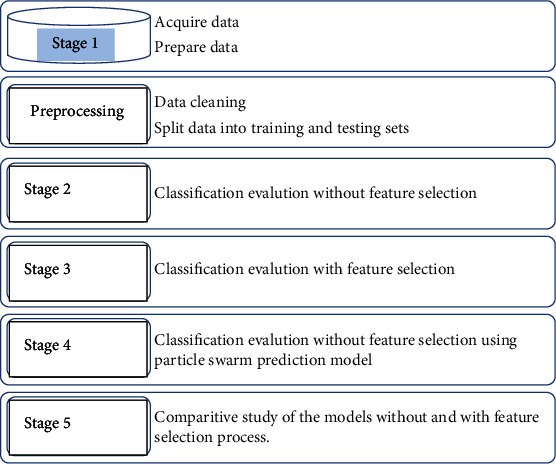
The proposed model.

The research model for this study is based on knowledge discovery technique and is depicted in Figure 2. The dataset preparation stage is the first one of the model, in which we acquire the applicable data for the research. The second stage is the preprocessing phase, in which the data is cleansed, and converted to be fit for the classification process and the feature extraction process. The processed data are then passed to stage 3 for classification. In stage 4, the proposed grasshopper prediction model is trained and validated using *k*-fold validation technique. The final stage is a comparative study of the models without and with feature selection process.

### 3.1. The Detailed Description of the Propose Model

The following subsections will describe the five stages of the proposed model.

#### 3.1.1. Stage 1: Data Selection

In the data collection stage, we acquired the cervical cancer data from two public datasets [24, 25]. The Cervical Cancer Prognostic Dataset in [24] has 1614 while the dataset in [25] has 1500 images. All images in both datasets were labeled by medical experts. The images are captured with resolution of 0.197 *μ*m/pixel. The images were manually classified by medical experts into seven classes. Images are partitioned into two parts: the cytoplasm and the nucleus, after subtracting the background. The partitioning was validated by medical experts for better accuracy.

#### 3.1.2. Stage 2: Preprocessing

This stage has data cleansing and partitioning subphases as depicted below.

*(1) Data Cleansing*. The acquired dataset will go via data cleansing technique. To clean noisy data, the records with unfitting data attributes are eradicated. Also, inconsistency in the data format will be controlled at this stage.

*(2) Data Partitioning*. In the data partitioning stage, we parted the data into two sets: the training and testing sets. We divided the dataset into 70% training and 30% testing. The partitioning of the data guarantees that the results are not over fitted during the testing phase.

#### 3.1.3. Stage 3: Classification without Feature Selection

In this stage, we built two models based on the fuzzy *C*-means with locality fitting model [29] and *C*-means [21–23], with 12-fold classifier crossvalidation. The training set with all the extracted feature was utilized for evaluation.

#### 3.1.4. Stage 4: Classification with Feature Selection

Features are selected by the QGH algorithm to select the best features. Using these features, we will build two models based also on naïve CNN model and *C*-means.

#### 3.1.5. Stage 5: Comparison and Analysis

In this stage, we performed comparison of the two models with and without the QGH feature selection. We examined the four models for overfitting. Examining of the prediction fitness is performed using confusion matrix [31–33], which include information about labeled actual and predicted classes as gotten by the classifier.

The model is tested with benchmark dataset validation under supervised learning to validate the correctness and accuracy of the prediction model. The metrics considered for efficiency are classification accuracy. The experiments are evaluated by measuring sensitivity, recall, specificity, and ROC curves [34, 35]. The proposed model is tested in comparison to existing similar models for accuracy and efficiency.

In this stage, we validate the experimental results of different classifier models with and without utilizing QGH feature selection. The comparison is directed to measure prediction model correctness, precision, and statistical measure.

### 3.2. Feature Selection Algorithm Description

Classification can encompass only relevant features to make the classification cost-effective in terms of computational power and time. Therefore, we recommend the methods that select the relevant features. Prior feature selection enhances classification time and reduces the computation workload. Also, Prior feature selection increases accuracy and precision. The proposed quantum-based grasshopper computing algorithm reveals more accurate feature selection with less computational load.

The advantages of QGH for feature selection are as depicted as follows:

- It has an influential exploration to reach the optimal solution where dissimilar particles will search in the solution space
- It incorporates grasshopper memory which is efficient for feature selection. Prior solutions are remembered by all grasshoppers as they hover in the solution space
- It requires less computational load because of the fast quantum computing
- It employs population of possible solutions instead of a single solution
- It can deal with binary and nonbinary data
- It requires less memory and computational time that uses simple mathematical operators with few parameters and unpretentious to the problem dimension

The algorithm of the QGH algorithm is depicted below:

### 3.3. Fuzzy *C*-Means and the Quantum Approach for Feature Selection and Cell Classification

Fuzzy *C*-means is an extended version of the standard *C*-means algorithm but with fuzzy integration [31]. Fuzzy intuition is utilized to generalize the *C*-means values of the members in each class.

Pap smear test can classify a cell into several classes including superficial and intermediate squamous as well as mild, moderate, and severe dysplasia. Also columnar and carcinoma are identified by the pear smear test. The accuracy of manual cell type detection prevails inaccurate classification as well as long diagnosis time. Pap smear images contain several features such as shape, color, and texture. Accurate feature extraction from this visual content is very critical in evolving an automated cervical cancer screening. The proposed technique fuses the feature extraction process with the *C*-means algorithm to select the most suitable features for classification of cancerous cells in Pap smear test.

In the first phase of the proposed method, all features significant to the color, geometric shape, or texture of the Pap smear are selected. In the second phase, the proposed deep learning algorithm with the clustering algorithm is utilized for feature selection and cancer classification. We can achieve accurate cell classification through the feature extraction phase of the thirteen features of geometric features as well as texture features of the Pap smeared images. These features are depicted as follows:

Here are the following for the rectangle surrounding the nucleus:

1. Area, *A*_*n*_ the number of pixels
2. Length, *L*_*n*_
3. Width, *W*_*n*_
4. Aspect ratio AR = *w*_*n*_/*l*_*n*_
5. Perimeter, *P*_*n*_
6. Nucleus roundness, *N*_circle_
7. Homogeneity of nucleus using histogram
8. Brightness, *B*_*n*_ = the mean intensity of pixels

Here are the following for the cytoplasm:

1. Maximum value of pixels in the cytoplasm region, Max*_c_*
2. Minima value of pixels in the cytoplasm region, Min*_c_*
3. Brightness: *B*_*c*_ which is the average pixel intensity

Here are the following for the cell region:

1. Number of pixels
2. Ratio of area of the nucleus to the area of the cell

Texture features can be extracted from the pap smeared images by utilizing the binary histogram Fourier algorithm (BHF). The algorithm starts by using the operator BHF to find the patterns among the data and compute the histogram. The next step is to use discrete Fourier transform (DFT) and compute it from the computed histogram. The final phase is to compute the feature vector by defining histogram zero values as well as all ones and Fourier spectrum coefficients. Based on the BHF and the defined features *a* to *k* above, we can define a feature vector of thirteen entries. The feature selection phase of our proposed method combines two units, namely, the QGH unit and the fuzzy *C*-means unit. The two units improve the accuracy of the classification procedure in Pap smeared cell images.

The fuzzy *C*-means is implemented before the computation of the fitness function. The QGH algorithm is utilized to obtain the variation of the features subset of the particles that are updated by computing the fitness values from the F1 score. The next phase is applying the fuzzy *C*-means to classify the smear images classes. The particles that attain the best fitness will be taken as the local best location and the global best location and have the best subset features. In this paper, we utilize the fuzzy *C*-means to improve the cell classification accuracy in the Pap smeared images and attain the best location of the particle.

## 4. Similar State-of-the Art Classification and Feature Selection Algorithm Description

In this section, we are going to describe the classification and feature selection algorithms.

### 4.1. Classification Description

In this research, two classification techniques, CNN model and C-Means, are tested in the classification of cervical cancer by using the prognostic cervical cancer dataset and compared to our model. The following subsections describe both techniques.

#### 4.1.1. CNN Model

CNN model-based classifiers define a supervised statistical learning technique. They employ probabilistic computational models and detect uncertainty by finding the probabilities of the outputs. These classifiers can aid in automated diagnosis. CNN model-based classifiers offer learning techniques that combines prior transfer knowledge and actual observations. CNN model-based classifiers build a useful viewpoint for evaluating many supervised learning techniques. It computes probabilities for a proposition and is characterized by their robustness against noise.

#### 4.1.2. *C*-Means Technique

*C*-means technique is a supervised learning classification technique. The neighbors of the target point are selected, by choosing minimum similarity metric such as Euclidean distance metric [36]. To predict the class of a new unknown instance, the *C*-means model will figure the distance to all labeled instances and states the nearest neighbors and their particular labels. The unknown new instance is classified by majority voting.

## 5. Experiments

The hybrid methodology that was presented in Figure 2 will be validated through experiments regarding the implementation of our proposed technique, for feature selection in Pap smeared image classification.

### 5.1. The Dataset

**Figure 3:**
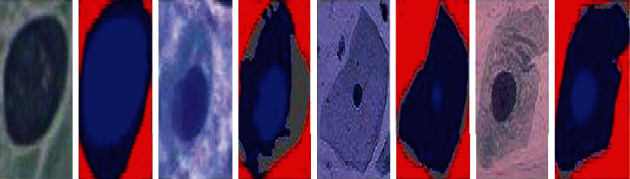
Images from the datasets [24, 25].

For our experiments, the Pap smeared single cell images from two dataset in [24, 25] will be utilized as our dataset. The first dataset has 1614 images, and the second one has 1500 images that were labeled by medical experts. The images are captured with resolution of 0.197 *μ*m/pixel. The images were manually classified by medical experts into seven classes. Images are partitioned into two parts: the cytoplasm and the nucleus, after subtracting the background. The partitioning was validated by medical experts for better accuracy. Some images from the datasets along with their labels are shown in Figure 3, and description of the dataset and images categories and distribution is depicted in Table 1.

### 5.2. Evaluation Results

#### 5.2.1. The Evaluation of the Proposed Quantum-Based Grasshopper Computing Algorithm

Different techniques are used for performance evaluation for image classification. Precision is computed as the number of true positives divided by the number of images classified by the system as positive. Recall is defined as the number of true positives divided by the actual number of positive samples in the dataset. F1 score combines both precision and recall [32–35].

We used the *K*-fold validation method in the experiments to be suitable for our dataset size. The seven features that were pruned from the thirteen features include three features for the nucleus, namely, the area, roundness, and brightness. The cytoplasm is characterized by the brightness feature. The cell is featured by its entire area. We utilized also the ratio of nucleus to the cytoplasm and the binary histogram Fourier algorithm (BHF) [35–38]. Our experiments are establishing the importance of the feature selection on the accuracy of the proposed classifier.

We devised two scenarios for the experiments: the first experiment, we made a comparison of the classification accuracy using feature selection versus classification without previous feature selection. Classification without previous feature selection means we use all the features. The experimental results are shown in Table 2. Using previous feature selection has enhanced the classification accuracy as compared to experiments that utilizes all the features.

In the second scenario, we detect the impact of previous feature selection on the results accuracy for different cervical cells in the Pap smears as shown in Table 3. Better accuracy is detected in previous cell selection than with all-feature approach.

In conclusion of our results, we tested our objectives of the importance of the integration of quantum-ness into our models. As reported it improved the accuracy of all the classifier methods including Fuzzy C-Means. Also, the accuracy recorded of the proposed approach is due to the rationality of the Fuzzy C-Means and how it improved the search capability of the QGH algorithm.

#### 5.2.2. Comparison of Classifiers Using CNN Model and *C*-Means with and without Feature Selection versus our Proposed Model

We built two prediction evaluations utilizing the training set for both CNN model and *C*-means algorithms. The twelvefold validation model is employed as validation of the models. The models apply all the features enclosed in the prognostic Pap smear dataset without no prior feature selection.

The CNN architecture encompasses a convolution layer, an RELU activation layer, and pooling layers of 3 × 3 and 1 × 1 sizes. In the training phase, this CNN is utilized to extract features and build the feature maps. The CNN architecture is depicted in Table 4.

**Figure 4:**
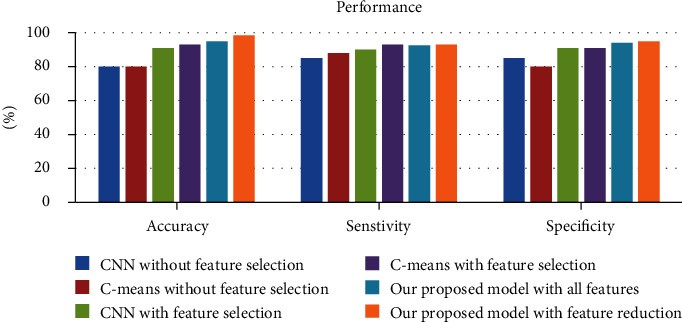
The comparison evaluation.

The evaluation results are described in Figure 4 depicting the performance of both models versus our model.  Table 5 demonstrates the statistics for the two models.  Table 6 depicts a confusion matrix of the accuracy, specificity, and sensitivity for the CNN model classifier and the C-Means algorithm.

#### 5.2.3. Discussion of Performance Results

In the experiments evaluation, we utilized accuracy, specificity, and sensitivity performance measures. The experimental results demonstrate that by employing feature selection, the three classifiers accomplished better accuracy than the same classifiers without feature selection. The best accuracy level for cervical cancer detection was achieved by our proposed PQSO classifier, which obtained 98% accuracy outperforming CNN model and *C*-means classifiers.

**Figure 5:**
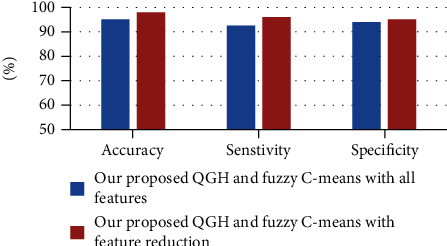
Accuracy results for proper feature space reduction.

The experiments with feature selection, also the experiments designated the proper feature space reduction of the dataset, can enhance the results by a reasonable margin. Accuracy results of these cases are depicted in Figure 5.

**Figure 6:**
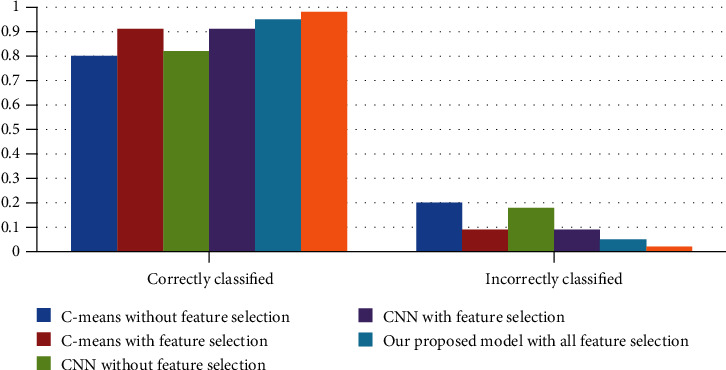
Correctly classified versus incorrectly classified instances.

Figure 6 displays correctly classified versus incorrectly classified instances. The results show improvement with feature selection and improved better with feature reduction for our model. This implies that feature reduction increases accuracy quantitatively and qualitatively because it concentrates only on relevant features. Of the three models, our proposed model achieved the utmost improvement with feature reduction.

Comparison of the classification time in seconds was performed and executed on a GPU GTX 1040 system. The presented comparison study demonstrates the classifying Pap smear images with the same training dataset. Our proposed model with the prior seven feature reduction QGH algorithm with fuzzy *C*-means is the fastest algorithm by an order of magnitude of 2, followed by QGH with all features accounted. The CNN model classifier is the next in classification time with feature selection still slower by an order of magnitude 2. During all experiments, the slowest is the *C*-means classifier. The comparison is depicted in Table 7.

## 6. Concluding Remarks

Improving classification accuracy is very important for cervical cell detection in Pap smear images. To improve the accuracy, our study presented a hybrid feature selection algorithm that incorporates the quantum-based algorithm (QGH) algorithm with the fuzzy *C*-means algorithm. From thirteen features that present shape, color, and texture of the Pap smear images, the QGH is utilized to prune the unimportant features down to collection of the best seven features.

The seven features that were pruned from the thirteen features are three features for the nucleus, namely, the area, roundness, and brightness. The cytoplasm is characterized by the brightness feature. The cell is featured by its entire area. We utilized also the ratio of nucleus to the cytoplasm and the binary histogram Fourier algorithm (BHF). Our experiments established the importance of the feature selection on the accuracy of the proposed classifier after we run two scenarios of the experiments: the first one, we made a comparison of the classification accuracy using feature selection versus classification without previous feature selection.

## Figures and Tables

**Algorithm 1:**
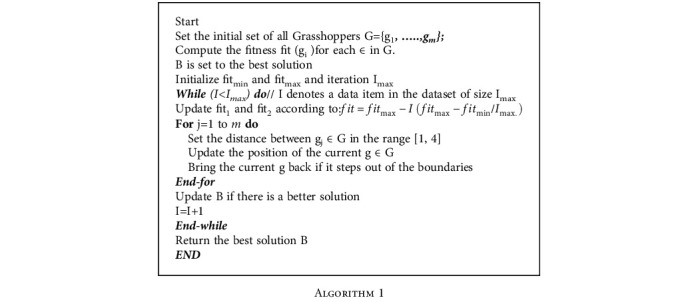


**Table 1:** Image categories and distribution in datasets [24, 25].

Cell	Category	Number of images in [24]	Number of images in [25]
Normal	Preliminary squamous	174	190
	Moderate squamous	170	250
	Columnar	190	280

Cancerous	In situ	258	240
	Preliminary dysplastic	280	290
	Intermediate dysplastic	248	100
	Last stage dysplastic	294	150

Total		1614	1500

**Table 2:** Comparison of results for experiment 1 with different numbers of clusters (*k*).

k	Without feature selection			Thirteen features			Seven features		
	Precision	Recall	F1-measure	Precision	Recall	F1-measure	Precision	Recall	F1-measure
3	0.77	0.74	0.70	0.73	0.80	0.76	0.73	0.79	0.76
4	0.77	0.79	0.77	0.81	0.82	0.83	0.87	0.88	0.89
5	0.74	0.74	0.74	0.85	0.84	0.86	0.95	0.96	0.95
6	0.76	0.76	0.76	0.87	0.84	0.87	0.93	0.91	0.93
7	0.73	0.73	0.73	0.84	0.86	0.85	0.94	0.95	0.94
8	0.79	0.70	0.79	0.81	0.82	0.81	0.93	0.94	0.93

**Table 3:** Impact of classification accuracy using previous feature selection versus all features on different cervical cells.

Cell category	All features			Previous 13 features			Previous 7 features		
	Precision	Recall	*F*-score	Precision	Recall	*F*-score	Precision	Recall	*F*-score
Normal	0.87	0.87	0.84	0.95	0.94	0.97	0.98	0.95	0.95
Intermediate	0.85	0.74	0.78	0.89	0.84	0.87	0.98	0.96	0.96
Columnar	0.77	0.77	0.75	0.95	0.95	0.98	0.99	0.94	0.99
In situ	0.74	0.87	0.78	0.94	0.97	0.94	0.98	0.90	0.97
Preliminary dysplastic	0.87	0.78	0.84	0.99	0.94	0.94	0.984	0.97	0.97
Intermediate dysplastic	0.79	0.85	0.77	0.87	0.97	0.94	0.986	0.97	0.97
Final stage dysplastic	0.79	0.75	0.70	0.87	0.95	0.94	0.988	0.94	0.95

**Table 4:** CNN architecture describing network layers.

Layer number	Network layer	Description
1	Input layer	Size of input image: 512 × 512 × 3
2	Convolutional kernel layer	256 × 8 × 1 convolutions
3	Pooling layer	Average pooling
4	Convolutional kernel layer	64 (5 × 5 × 3) convolutions
5	Pooling layer	Max function pooling
6	Convolutional kernel layer	32 (3 × 3 × 3)
7	Fully connected	1048 neurons
8	Softmax classifier	

**Table 5:** Statistics for using CNN model and *C*-means with and without feature selection and our QGH algorithm with fuzzy *C*-means.

	CNN model classifier without feature selection	CNN model classifier with feature selection	*C*-means classifier without feature selection	*C*-means classifier with feature selection	Our proposed model with prior 7 feature selection QGH algorithm with fuzzy *C*-means
Correctly classified	0.841	0.912	0.867	0.919	0.96
Incorrectly classified	0.159	0.088	0.137	0.081	0.04
Kappa coefficient (interqualitative reliability)	0.187	0.201	0.177	0.299	0.314
Mean square error	0.841	0.521	0.648	0.483	0.311

**Table 6:** Confusion matrix for our proposed QGH with fuzzy *C*-means using 30% of both dataset in [24, 25] (900 test cases).

		Predicted cases	
		Positive	Negative
Actual cases	Positive	482	30
	Negative	6	382

**Table 7:** Comparison of the classification time in seconds.

Method	Execution time (sec)
Our proposed model with the prior 7 feature reduction QGH algorithm with fuzzy *C*-means	4.42 ± 0.115
Our proposed model with all feature selection QGH algorithm with fuzzy *C*-means	9.25 ± 0.429
CNN model with prior 7 feature reduction	140.42 ± 0.329
CNN model with all feature selection	290.32 ± 0.223
*C*-means with prior 7 feature reduction	790.29 ± 0.929
*C*-means with all feature selection	1290.42 ± 1.029

## Data Availability

The data is available in http://mde-lab.aegean.gr/.
